# Measuring Health Literacy in Romania: Validation of the HLS-EU-Q16 Survey Questionnaire

**DOI:** 10.3389/ijph.2022.1604272

**Published:** 2022-02-04

**Authors:** Madalina Adina Coman, Alina Ioana Forray, Stephan Van den Broucke, Razvan Mircea Chereches

**Affiliations:** ^1^ Department of Public Health, Faculty of Political, Administrative and Communication Sciences, Babeș-Bolyai University, Cluj-Napoca, Romania; ^2^ Psychological Sciences Research Institute, Catholic University of Louvain, Louvain-la-Neuve, Belgium

**Keywords:** health literacy, HLS-EU-Q16, Romania, measurement, validation

## Abstract

**Objective:** Empirical data on health literacy (HL) for Romania is almost inexistent. The present study aimed to validate the HLS-EU-Q16 questionnaire for the Romanian population and explore the predictors of HL in the North-West Region of the country.

**Methods:** A non-experimental, cross-sectional study was conducted between March and November 2019 on a representative, stratified random sample of 1,622 participants from the North-Western Region of Romania. Exploratory Factor Analysis (EFA), Principal component analysis (PCA), Pearson correlations, and Chronbach’s Alpha were used to validate the scale. Linear regressions were conducted to assess the determinants of health literacy.

**Results:** Results obtained for the HL scale support its factorial component and reliability, with a Cronbach’s alpha of *α* = 0.84. Age, gender, education and self-reported health status were identified as determinants of HL.

**Conclusion:** Study findings indicate that the Romanian version of HLS-EU-Q16 is psychometrically sound and comparable to the original version. These results provide the first validated tool to measure HL in Romanian and the first population level data for Romania.

## Introduction

Health literacy (HL) is a concept that is growing and attracting interest at international level. Originally introduced in the 1970s to refer to patients’ understanding of information in a medical context, the concept gradually expanded in meaning to also account for more complex and interconnected abilities, such as reading and acting upon written health information, communicating needs to health professionals, and understanding health instructions, thus finding its way into public health. Research shows that low levels of HL lead to overall poorer health, more hospitalizations, greater use of emergency services, low treatment compliance and adherence, poor ability to communicate with the medical provider and understand medical and health information, poorer vaccine compliance, and poorer adherence to screening procedures [1, 2].

With the growing recognition of the impact of limited HL, initiatives to address limited HL emerged in North America, Australia, and Europe. While the measurement of HL in the United States focused mostly on clinical HL [3], the direction taken to measuring HL in Europe took another approach, starting from a broad concept of HL that focused on the general population through the European Health Literacy Survey (HLS-EU) Consortium. The first step was thus to conduct a rigorous literature review to explore and define health literacy [4]. This resulted in a comprehensive, consensual definition of HL stating that: “health literacy is closely linked to literacy and entails people’s knowledge, motivation and competencies to access understand, appraise and apply information to form judgements and to make decisions in everyday life concerning healthcare, disease prevention and health promotion, to maintain and improve quality of life during the life course” [4]. This definition covers the concept of HL from both an individual and a systemic perspective. Importantly, it relates not only to understanding health related information, but also to finding/accessing, evaluating/appraising and using/applying that information to make decisions on health. In this way, it relates to the typology of functional, interactive and critical health literacy that was proposed by Nutbeam [5], whereby finding/accessing information refers to interactive health literacy, understanding to functional health literacy, and evaluating/appraising information to critical health literacy.

Based on this inclusive definition, the HLS-EU Consortium developed a comprehensive self-report questionnaire to measure HL in three broad domains of health (healthcare, disease prevention, health promotion), incorporating four cognitive information-processing competencies (obtaining, understanding, evaluating, and using health information). The questionnaire, named HLS-EU-Q47, consists of 47 items in the form of questions regarding the ease of processing health information, to be scored on a 4-point Likert scales. As the 47-item questionnaire is rather long when used in combination with other instruments, two short versions of the questionnaire were also created: a 16-item version (HLS-EU-Q16), and a 6-item very short version (HLS-EU-Q6). Both these short forms were created using item response theory and Rasch analysis [6, 7], implying that the psychometric properties looked for were mainly those of a one-dimensional scale while ensuring that the underlying scope and theoretical concept of the extended form were sufficiently represented [7, 8].

While the original HLS-EU survey was applied in eight European countries (Austria, Bulgaria, Germany, Greece, Ireland, Netherlands, Poland, and Spain), similar surveys using the HLS-EU-Q47, HLS-EU-Q16 or HLS-EU-Q6 have been conducted in a number of other countries worldwide, including Belgium, Czech Republic, Denmark, France, Hungary, Italy, Malta, Portugal, India, Indonesia, Israel, Japan, Kazakhstan, Malaysia, Myanmar, Taiwan and Vietnam. Although levels of HL vary widely between countries and between groups within countries based on gender, education level or socio-economic status, the overall pattern is that in many countries up to half of the population, and sometimes more, presents with limited or insufficient levels of HL [9–12].

Contrary to the growing interest in HL in many European countries and despite its importance for the health care and public health sectors, official data on HL in Romania is almost inexistent, as shown by previous research [13, 14]. In Romania, to our best knowledge, there is only one study assessing health literacy, focusing on the HL of the general population in the rural area of Cluj county [15]. Other published research on HL in Romania focuses on maternal HL among Hungarian mothers [16], mental HL of mothers [17], and adult literacy in dentistry [18]. Thus, it appears that research on HL in Romania is not only scarce, but also not aligned with the standards developed at EU level. There is no national study assessing the level of HL of the general population, and policies concerning HL are yet to be developed. Since policy efforts should begin with assessing and addressing the HL needs in the general population, the present study aimed to validate the HLS-EU-Q16 survey for the Romanian population and to explore the predictors of HL in a sample of population from the North-West Region of Romania.

## Methods

### Study Design, Setting, Participants

A non-experimental, cross-sectional study using a survey was conducted during weekends between March and November 2019 on a sample of 1,622 participants from four counties from the North-Western Region of Romania. Participants were a representative, stratified random sample from the North-West region of Romania, selected following the rules of probabilistic sampling of the electoral lists, proportionally with the size of randomly selected localities from the Romanian counties of Bihor, Sălaj, Bistrița-Năsăud, Maramureș and Cluj. Within the five regions, 43 cities and villages were randomly selected using the website random.org, with the probability of selection being proportional to the size of the population. The sampling points were distributed proportionately with the population distribution—the polling sites were initially selected, then the starting streets at each polling site. Within each sampling point, a proportional number of households were systematically randomly selected. Starting from each sampling point, a random number was selected for each street as a starting point. After the initial assessment of each starting point, every 5th address was selected for the completion of one survey, until the required number of questionnaires for that place was reached. The eligibility criteria for participants were: 18 years or above, Romanian residency in the North-Western Region and Romanian language, no clear sign of psychological or learning disabilities, and willingness to answer the survey.

### Data Collection

The survey was completed using a mobile device by 12 trained and paid surveyors. The Survey Monkey platform was used for data collection and survey form filling. In the areas where no mobile reception was available, the surveyors used the pen-paper version of the survey and they later inputted it onto the electronic platform. Before going into the field, the surveyors attended a training session designed to cover the Standard Operating Procedure (SOP) and different situations that might appear while on the field. A data collection supervisor was present each weekend at the data collection site and offered support for data collection.

The data collection process was initiated after obtaining all the ethics approvals. Before being enrolled in the study, all participants signed two informed consent forms (one of them remained to the respondent, the other one was handed to the surveyor). The response rate to the survey was 72.63%.

### Instrument

The survey was part of a larger study that assessed HL, food literacy, nutrition’s impact on health, usability and understanding of nutrition labels, eating behavior, sugar consumption, soy consumption behavior and beliefs, and demographics. For this study, only variables related to HL and demographics (age, gender, residence, education, occupation, living conditions, marital status) were used.

Health literacy was assessed using the European Health Literacy Survey Questionnaire, short version with 16 items (HLS-EU-Q16) developed by the HLS-EU Consortium [6, 7, 11]. This tool was chosen for translation and validation as it is a short and easy to administer instrument designed for the general population rather than for specific patient groups. The tool measures general health literacy considering its four core competencies (find, understand, appraise and apply health information) in three domains (health care, disease prevention, health promotion) [6–8, 11]. Each of the 16 items are scored on a 4-point Likert scale ranging from 1 (“very difficult”) to 4 (“very easy”). In accordance with the guidelines for the original HLS-EU-Q16, the responses are dichotomized whereby “very difficult” and “fairly difficult” become “difficult” (scored with 0) and “very easy” and “fairly easy” become “easy” (scored with 1). For interpretation of the final score, all items of the scale are summed, giving a score ranging between 0 (no health literacy) to 16 (high health literacy). For ease of interpretability, the three levels are identified: inadequate health literacy (0–8), problematic health literacy [9–12], and sufficient health literacy [11, 13–16]. For the linear regression model performed in this study, the raw scores to the scale were used.

A systematic approach to translate and adapt the used instruments for the larger study was conducted, following the guidelines of the World Health Organization [19]. This systematic process involved five steps: forward-translation, expert panel discussion, back-ward translation, a pre-test, a cognitive briefing, and a consensus on the final version. Two translators with Romanian as mother tongue translated the items into Romanian. The translations were combined into a Romanian version that was further discussed by an expert panel with public health knowledge. Back-translation was done by another researcher who did not have knowledge of the survey beforehand. The translated version was compared to the original HLS-EU-Q16 instrument. Minor discrepancies were addressed based on consensus in the expert panel. Next, the questionnaire was pre-tested on 100 respondents to investigate the level of comprehensibility and cognitive equivalence of the translation, using face-to-face and telephone survey interviews. This data was solely for the cognitive interview process and was not used in the final analysis. After the cognitive briefing, the consensus on the final version included minor adaptations, such as offering a short definition of mental health for items 8 and 13 and a brief description for media outlets for items 11 and 15. Another scale adaptation was adjusting the pronouns of items to a more formal version in the Romanian language.

### Data Analysis

Data analyses were conducted using SPSS Statistical Software version 21 and Monte Carlo PCA for Parallel Analysis online version [20]. Descriptive statistics were conducted to describe and explore the sample. Exploratory Factor Analysis (EFA), Principal component analysis (PCA), Pearson correlations, and Chronbach’s Alpha were used to assess the reliability of the health literacy scale. Linear regressions were conducted to assess the associations between health literacy and socio-demographics. To determine the construct validity of the HLS-EU-Q16 for the Romanian population a Principal Component Analysis (PCA) with an Oblimin rotation was performed on all 16 items, as suggested in other validation studies [21]. Results of a preceding EFA showed a Kaiser-Meyer-Oklin (KMO) value of 0.850, supporting the sampling adequacy for the analysis. Bartlett’s test of sphericity (χ2 = 9045.820, df = 120, *p* < 0.001) indicated that correlations did not occur by chance, and that they were strong enough for the analysis. In order to identify the predictors of HL, a linear regression model was conducted with the score on the HL scale [1–16] as dependent variable, and age, gender, level of education, self-reported health and residence as independent variables. For the HL score a low score was associated with a low level of HL and a high score with a high level.

## Results

A total of 1,715 participants completed the survey. Following the exclusion of 93 questionnaires for which the responses on the HLS-EU-Q16 were incomplete, a final sample of 1622 participants was retained. Table 1 presents the demographics of the sample and shows that most of the participants in the sample were females (61.2%), and that slightly more than half lived in the rural area (52.1%). Most respondents considered that overall, their health was good (43.3%) with only 14.8% stating that they had a bad or very bad health status.

**TABLE 1:** Sample description (n = 1622). Functional Collaboration Model between Public Research Organizations and the Economic Environment for the Delivery of High-level Scientific and Technological Services in Bioeconomy Project, Romania, 2018-2021.

		N	%
Gender	Male	608	37.5
	Female	992	61.2
	*Missing cases*	*22*	*1.2*
Residence	Rural	845	52.1
	Urban	752	46.4
	*Missing cases*	*25*	*1.5*
Education	No formal education	9	0.6
	Primary school	71	4.4
	Middle school	264	16.3
	High school or equivalent	715	44.1
	University or equivalent	488	30.1
	*Missing cases*	*75*	*4.6*
Occupation	Employed	717	44.2
	Unemployed looking for job	47	2.9
	Retired or social welfare	680	41.9
	Student	65	4.0
	Homemaker	87	5.4
	*Missing cases*	*26*	*1.6*
Marital status	Married	1088	67.1
	Not married	179	11.0
	Divorced	48	3.0
	Widow	232	14.3
	Single	32	2.0
	Living with partner	13	0.8
	*Missing cases*	*44*	*2.7*
Living conditions	Alone	259	16.0
	Alone with children	88	5.4
	Couple without children	502	30.9
	Couple with children	469	28.9
	Sharing place with others	139	8.6
	Adult living with parents	95	5.9
	*Missing cases*	*70*	*4.4*
Have children	Yes	1296	79.9
	No	304	18.7
	*Missing cases*	*22*	*1.4*
Overall health	Excellent	120	7.4
	Good	703	43.3
	Moderate	556	34.3
	Bad	205	12.6
	Very bad	36	2.2
	*Missing cases*	*2*	*0.1*
Health information ranking	Total lack of information	15	0.9
	2	33	2.0
	3	50	3.1
	4	84	5.2
	Quite informed	296	18.2
	6	186	11.5
	7	276	17.0
	8	353	21.8
	9	191	11.8
	Very well informed	131	8.1
	*Missing cases*	*7*	*0.4*
Age	Mean	SD	
	53.53	17.48	

SWEETCONOMY Project, Romania, 2018–2021.

### Health Literacy

Table 2 shows the responses for the individual items of the HLS-EU-Q16. The results reveal that a very large percentage (21.6%) of the respondents found it very difficult to protect themselves from illness based on the health information from the media (Q12). Relatively high percentages of “very difficult” answers are also observed for other questions relating to assessing health information from the media (Q11, Q15), as well as relating to mental health issues (Q8, Q13). On the other hand, high percentages of respondents declared to find it very easy to understand why health screenings are needed (Q10), to understand health warnings about different behaviors (Q9), and to understand the doctor’s and pharmacist’s instructions (Q4). The majority of the items of the HLS-EU-Q16 instrument were significantly correlated at *p* < 0.01, as shown in Table 3.

**TABLE 2:** Health literacy survey responses. Functional Collaboration Model between Public Research Organizations and the Economic Environment for the Delivery of High-level Scientific and Technological Services in Bioeconomy Project, Romania, 2018-2021.

On a scale from very easy to very difficult, how easy would you say it is to…	Very difficult	Fairly difficult	Fairly easy	Very easy
	%	%	%	%
1. Find information on treatments of illnesses that concern you?	3.1	15.5	51.3	30.1
2. Find out where to get professional help when you are ill?	2.5	14.7	44.2	38.6
3. Understand what your doctor says to you?	1	9.8	46.7	42.4
4. Understand your doctor’s or pharmacist’s instruction on how to take a prescribed medicine?	0.6	4.7	46.2	48.5
5. Judge when you may need to get a second opinion from another doctor?	3.5	20.2	45.3	31.1
6. Use information the doctor gives you to make decisions about your illness?	0.5	5.7	50.4	43.4
7. Follow instructions from your doctor or pharmacist?	0.7	7.2	44.9	47.2
8. Find information on how to manage mental health problems like stress or depression?	5.5	25.0	43.8	25.6
9. Understand health warnings about behavior such as smoking, low physical activity and drinking too much?	0.7	5.7	37.8	55.8
10. Understand why you need health screenings?	0.9	6.3	34.2	58.7
11. Judge if the information on health risks in the media is reliable?	7.6	31.1	40.3	21.0
12. Decide how you can protect yourself from illness based on information in the media?	21.6	43.0	25.2	10.3
13. Find out about activities that are good for your mental well-being?	5.9	26.1	44.1	23.8
14. Understand advice on health from family members or friends?	4.4	15.8	46.4	33.3
15. Understand information in the media on how to get healthier?	7.3	25.4	46.0	21.3
16. Judge which everyday behavior is related to your health?	0.9	9.2	52.4	37.4

SWEETCONOMY Project, Romania, 2018-2021.

**TABLE 3:** Pearson correlation analysis of the health literacy items. Functional Collaboration Model between Public Research Organizations and the Economic Environment for the Delivery of High-level Scientific and Technological Services in Bioeconomy Project, Romania, 2018-2021.

	1	2	3	4	5	6	7	8	9	10	11	12	13	14	15
2	0.517**														
3	0.413**	0.445**													
4	0.374**	0.357**	0.628**												
5	0.283**	0.204**	0.316**	0.381**											
6	0.336**	0.336**	0.500**	0.558**	0.443**										
7	0.327**	0.351**	0.483**	0.545**	0.389**	0.654**									
8	0.280**	0.166**	0.252**	0.280**	0.288**	0.279**	0.269**								
9	0.219**	0.198**	0.220**	0.254**	0.222**	0.292**	0.279**	0.300**							
10	0.256**	0.239**	0.324**	0.370**	0.319**	0.385**	0.373**	0.228**	0.429**						
11	0.214**	0.165**	0.166**	0.169**	0.225**	0.176**	0.181**	0.226**	0.212**	0.225**					
12	0.096**	0.049*	0.055*	0.012	0.107**	0.039	0.045	0.108**	0.043	0.056*	0.414**				
13	0.294**	0.166**	0.273**	0.292**	0.296**	0.305**	0.280**	0.804**	0.310**	0.222**	0.243**	0.127**			
14	0.126**	0.142**	0.109**	0.114**	0.086**	0.151**	0.181**	0.117**	0.192**	0.231**	0.198**	0.165**	0.081**		
15	0.218**	0.147**	0.140**	0.140**	0.173**	0.170**	0.138**	0.194**	0.210**	0.211**	0.517**	0.484**	0.197**	0.290**	
16	0.211**	0.158**	0.223**	0.282**	0.301**	0.336**	0.319**	0.244**	0.345**	0.369**	0.272**	0.142**	0.258**	0.311**	0.330**

*Correlation is significant at the 0.05 level (2-tailed).

**Correlation is significant at the 0.01 level (2-tailed)

SWEETCONOMY Project, Romania, 2018–2021.

After 7 iterations, a four-component model with eigenvalues exceeding 1 was obtained explaining 59.59% of the variance. The scree plot indicated an optimal model consisting of two to four components. A fit of a unidimensional and three component solution as developed by the original model [8], was explored first offering multiple factor loading values below 0.40. Therefore, a Monte Carlo PCA for Parallel Analysis was employed in order to determine the number of components to retain [22]. By systematically comparing the eigenvalues obtained during the PCA in SPSS with the results generated by the parallel analysis, all four components could be retained since all of them where larger than the eigenvalue randomly generated data matrix of the same size showing the best fit of the model (Table 4).

**TABLE 4:** Parallel analysis results. Functional Collaboration Model between Public Research Organizations and the Economic Environment for the Delivery of High-level Scientific and Technological Services in Bioeconomy Project, Romania, 2018-2021.

Component	PCA from SPSS	PCA from Monte Carlo	Decision
1	5.116	1.171	Accept
2	1.878	1.135	Accept
3	1.367	1.110	Accept
4	1.175	1.085	Accept

SWEETCONOMY Project, Romania, 2018–2021.

The item loading after rotation and the four components are illustrated in Table 5. The item loadings suggest that component 1 (items: 1, 2, 3, 4, 5, 6, 7) represent “Finding and Processing health care information”; component 2 (items: 11, 12, 15) represent “Processing and Using information from Media”; component 3 (items: 8, 12) represent “Finding and using information about mental health”; and component 4 (items: 9, 10, 14, 16) represent “Processing information in Connection to Prevention and Health Promotion.” The corrected item-total correlation for items was high as all items (except item 5) and received a correlation of 0.40 or higher with a range from 0.5 to 0.9. The results obtained for the HL scale support its factorial component and reliability, with a Cronbach’s alpha of *α* = 0.841, 95% CI (0.829, 0.852). Deleting any of the items from the scale did not significantly affect the Cronbach’s alpha, so the scale was kept as originally developed by the authors.

**TABLE 5:** Component loadings of the health literacy scale, after Oblimin rotation (n = 1622). Functional Collaboration Model between Public Research Organizations and the Economic Environment for the Delivery of High-level Scientific and Technological Services in Bioeconomy Project, Romania, 2018-2021.

	Components			
	1	2	3	4
1. Find information on treatments of illnesses that concern you?	0.664			
2. Find out where to get professional help when you are ill?	0.769			
3. Understand what your doctor says to you?	0.803			
4. Understand your doctor’s or pharmacist’s instruction on how to take a prescribed medicine?	0.724			
5. Judge when you may need to get a second opinion from another doctor?	0.373			
6. Use information the doctor gives you to make decisions about your illness?	0.625			
7. Follow instructions from your doctor or pharmacist?	0.625			
12. Decide how you can protect yourself from illness based on information in the media?		0.807		
15. Understand information in the media on how to get healthier?		0.756		
11. Judge if the information on health risks in the media is reliable?		0.692		
8. Find information on how to manage mental health problems like stress or depression?			−0.932	
13. Find out about activities that are good for your mental well-being?			−0.931	
9. Understand health warnings about behavior such as smoking, low physical activity and drinking too much?				−0.607
10. Understand why you need health screenings?				−0.641
14. Understand advice on health from family members or friends?				−0.587
16. Judge which everyday behavior is related to your health?				−0.673

SWEETCONOMY Project, Romania, 2018–2021.

When conversed into the three levels of HL, the majority of the participants in the sample (59.2%) attained a sufficient level of HL, 33.2% a problematic level, and 7.5% an inadequate level of HL.

### Predictors of Health Literacy

Results of the linear regression show a value of R^2^ = 0.115, meaning that the model explains 11.5% of the variance of HL. The F test was statistically significant (F = 39.775 *p* = 0.000), the model having a moderate explanatory power. Age, gender, education, and self-reported health are all significantly associated with health literacy. Place of residence was the only demographic variable not showing any association with health literacy (Table 6). Gender and education are positively associated with health literacy, while age and self-reported health are negatively associated with health literacy. Education had the strongest relationship with health literacy, followed by self-reported health, age and gender as the values of standardized coefficient Beta shows it.

**TABLE 6:** Linear regression model—health literacy and sociodemographic indicators. Functional Collaboration Model between Public Research Organizations and the Economic Environment for the Delivery of High-level Scientific and Technological Services in Bioeconomy Project, Romania, 2018-2021.

	Unstandardized coefficients		Standardized coefficient	95% CI for B			
	B	Std. Error	Beta	t	Sig	Lower	Upper
Gender	0.583	0.134	0.105	4.334	0.000	0.319	0.846
Age	−0.021	0.004	−0.129	−4.809	0.000	−0.029	−0.012
Education	0.360	0.049	0.189	7.403	0.000	0.264	0.455
Self-reported health	−0.538	0.079	−0.183	−6.768	0.000	−0.694	−0.382
Residence	0.174	0.134	0.032	1.300	0.194	−0.088	0.436

SWEETCONOMY Project, Romania, 2018–2021.

## Discussion

The aim of the study was to validate the HLS-EU-Q16 survey for the Romanian population and explore the predictors of HL in a sample of the Romanian population. The HLS-EU-Q16 for the Romanian showed good internal validity with a Cronbach’s alpha of *α* = 0.841. This is similar to the original scale [7] and to other validation studies of this survey conducted in Europe using EFA, Confirmatory Factor Analysis, and PCA, which reported values between *α* = 0.799, *α* = 0.982 [21, 23–28]. Similar to the Icelandic study, our scale identified four components [21] and similar to the Italian study, the same ceiling-floor effects were observed in the Romanian sample, showing that both Romanians and Italians find mental health information and health information as difficult to understand, process, appraise and use [23]. The only item that had a factor loading below 0.40 on any of the components resulting from the PCA was item 5. A possible explanation for this finding is that seeking for a second medical opinion is not a widespread practice in Romania yet [29].

The four components found in the Romanian sample do not represent the competencies of HL as given in the original model on which the questionnaire is based [8]. Except for the third component, which only included items representing the competency to find health related information, all dimensions represent more than one component of HL. This might indicate that finding health information is more salient for the Romanian population than the other steps in the health information processing cycle, as shown by the difficulty that is experienced when trying to understand, appraise and use information related to disease prevention and health promotion. On the other hand, the three domains of the HL model (health care, disease prevention, health promotion) are all represented in the structure resulting from the PCA, with component one including all items from the health care domain from the original questionnaire, while components two, three and four include items from both disease prevention and health promotion. The factor loading pattern found in this study is different from the original model [8] and from the patterns obtained in other studies [21], which is an indication that the domains of HL that underlie the questionnaire do not manifest themselves in the same way across cultures [10, 11].

While providing a valid instrument to measure HL, the study is also the first to provide population level data on HL in a Romanian population. To the best of our knowledge, there are no other studies of HL in the Romanian population using a validated health literacy scale. One previous study conducted in Romania focused on health literacy among rural inhabitants in Cluj County, but did not use a validated health literacy scale, only an adapted health literacy screener of eight questions measuring self-reported functional literacy skills that was aimed for patients and not the general population [15].

Our results show that most of the participants in our sample (59.2%) have a sufficient level of HL, while 33.2% have a problematic level and 7.5% an inadequate level of HL. These results are in line with results from studies conducted in Europe that used the HLS-EU-Q16 to measure HL in the general population. Slight differences can be observed regarding health literacy levels among European countries, African, and some Asian countries, with non-European countries reporting lower levels of adequate or sufficient HL. A study conducted in Italy showed that 11.8% of the sample presented inadequate HL, 55.2% problematic HL%, and 33.0% “enough” HL [23]. In Iceland, studies reported 72.5% people with sufficient HL, 22% with problematic HL and 5.5% with inadequate HL [21]. In Poland, results showed that “10.2% possessed inadequate general HL and 34.4% problematic HL, while 55.4% had a sufficient level of health literacy” [30]. Results from France reported 8% inadequate HL, 33% problematic HL, and 58% sufficient HL [24]. Results from a large study conducted in Denmark report 8.2% inadequate HL, 30.9% problematic HL and 60.9% sufficient HL [31]. Data from Israel reports that “more than 10% of the sample had inadequate health literacy, 21% had problematic health literacy, and 69% showed likely sufficient health literacy” [26]. In Ghana, studies showed that “only 34% of participants had sufficient HL while 28.1% had inadequate HL, and 37.9% presented problematic health literacy” [32]. Results from rural areas in Indonesia report that 63.5% of participants had inadequate or problematic health literacy, with only 36.5% reporting sufficient HL [33]. Similar results were reported in Ghana where in a sample of University students, 20.4% had inadequate HL and 34.2% had problematic HL [34] and in Spain where 68.7% Arabic migrants had a limited (inadequate and problematic) level of HL [35]. These differences can be explained by opportunities affecting the predictors of HL such as access to education, income, place of living and overall health with high-income countries having more opportunities as compared to Low- and Middle-income countries [36].

Our study identified age, gender, education, and self-reported health status as determinants of HL. Males, older people, people with lower levels of education, and people that rate their health as not very good have lower levels of HL. These results are in line with findings from other European studies on HL. Research shows that age is related to hearing or cognitive impairments, therefore it is more difficult for older patients to understand or judge more complicated aspects related to health [24]. Studies involving gender show that women have more health-seeking behaviors and are more involved in the health sphere, spending more time with doctors and having more medical encounters [37]. More years of education and better perceived health are strong predictors intently studied, which proved to influence the level of HL as well [38].

The results of our study in terms of predictors of HL are similar to results of the original study designing the instrument at the European level [11]. There are other studies from Europe and some countries in Asia that found the same predictors, or at least one of them, showing that age, gender, education and self-reported health are strong predictors of HL [6, 21, 23, 26, 30, 31, 35, 39]. Results from Ghana show similar results in term of self-reported health, age, education, but for gender they report higher levels of HL for men, contrary to our findings [32]. These results can be explained by the difference between some countries in Africa and Europe in terms of expectations and opportunities for men and women and reinforce the capability of the HLS-EU-Q16 tool to be adapted for different cultures.

The finding that residence area was not associated with HL, contrary to some other studies [40], can partly be explained by the cultural aspects of Romania. Health literacy is a dynamic construct, and it is the outcome of a combination of cognitive capacities, knowledge, opportunities, life experience and the context. As such, the cultural setting, which includes the quality of education, healthcare, welfare, as well as social and market systems, and the history of the country/region can all contribute to differences in HL between geographical areas. This study focused on the North-West Region of Romania, which tends to have high quality of education, good healthcare services, and very good economic growth [41]. For these reasons, local studies are further encouraged.

### Limitations

The current study has several limitations. Although the sample size of our study is representative for the North-West Region of Romania, an even larger sample would allow to expand the analysis at a national level. Our study was intended for the Romanian speaking population as a homogeneous group. In the future, a more comprehensive analysis could focus on the HL needs of smaller subgroups, such as different ethnic groups. In addition, due to the data collection procedure used, social desirability could not be excluded and may have resulted in some selection bias. Lastly, our study was cross-sectional, therefore no causality can be attributed to factors associated with health literacy.

The main strength of the study is the large sample which is representative for the entire North-West Region of Romania and the rigorous methodology for data collection using a random stratified sample. In addition, the study used an international validated tool, which allowed for a comparison of the results with those obtained in other European studies. In the longer term, this allows for a collaboration at EU and international levels, by inclusion the tool in the EU’s health reporting and monitoring system. Finally, the results of this survey can inform and support political and professional decision-making to improve HL.

### Conclusion

After translating and adapting the HLS-EU-Q16 to Romanian, the HLS-EU-Q16-RO is a valid instrument, ready to be used in Romania. The findings of the present study indicate that the Romanian version of HLS-EU-Q16 is psychometrically sound, with a reasonably clear four component structure, and comparable to the original model. The results of this study provide the first evidence for HL using the HLS-EU-Q16 survey and the first validated HL tool for Romania. Furthermore, these results can be used for future health education programs, promotion interventions and policy initiatives to improve HL and to support health systems to create health literate organizations in Romania.
